# Psychosocial resources and psychopathology among persons with neuromuscular disorders during the COVID-19 pandemic

**DOI:** 10.1186/s40359-024-01742-5

**Published:** 2024-04-29

**Authors:** Silvia Sanzo’, Federica Tizzoni, Stefano C. Previtali, Angela Berardinelli, Maria Nobile, Massimo Molteni, Martina Manzoni, Arianna Tarabelloni, Annamaria Russo, Antonella Delle Fave, Maria Grazia D’Angelo

**Affiliations:** 1 Child Psychopathology Unit, Scientific Institute IRCCS Eugenio Medea, Bosisio Parini, Italy; 2 Department of Pathophysiology and Transplantation, University of Milano, Milan, Italy; 3 Neuromuscular Repair Unit, Inspe and Division of Neuroscience, IRCCS San Raffaele Scientific Institute, Milan, Italy; 4 Child and Adolescence Neurology Unit, National Neurological Institute C. Mondino Foundation IRCCS, Pavia, Italy; 5 Unit of Rehabilitation of Rare Diseases of the Central and Peripheral Nervous System, Scientific Institute IRCCS Eugenio Medea, Bosisio Parini, Italy

**Keywords:** Inherited NeuroMuscular disorders (INMD), Resilience, Social Support, Psychopathology, COVID-19 pandemic

## Abstract

**Background:**

The COVID-19 pandemic substantially affected the lives of persons with inherited neuromuscular disorders (INMD), causing disruption in clinical and support services. While several studies have investigated mental health, distress and psychosocial resources in the general population during the pandemic, little is known about the experience of persons with INMD.

**Methods:**

This study was aimed to fill this gap by jointly investigating both psychopathological symptoms and psychosocial resources – specifically, resilience and perceived social support – among persons with INMD during the pandemic, taking into account demographic and clinical factors. Between April and December 2020, 59 participants with INMD (aged 15–59, 71.2% M) completed a questionnaire collecting demographic and clinical data, the Multidimensional Scale of Perceived Social Support, the Resilience Scale for Adults, and the Achenbach System of Empirically Based Assessment.

**Results:**

Overall, participants showed good levels of resilience and perceived social support. A minority of participants reported clinically relevant psychopathological symptoms, 28.81% for anxiety and depression. Most psychopathological symptoms were negatively correlated with resilience (-0.347 < *r* < − .420), but not significantly associated with social support. Consistent with previous studies, regression analyses highlighted that participants with Duchenne muscular dystrophy were more prone to report anxious and depressive symptoms (B = 1.748, *p* = .028, OR = 5.744), and participants with myotonic dystrophy, attention problems (B = 2.339, *p* = .006, OR = 10.376). Resilience emerged as a potential predictor of lower anxious-depressive symptoms (B=-1.264, *p* = .012, OR = 0.283).

**Conclusions:**

The findings suggest the importance to investigate psychosocial resources in addition to psychopathology among persons with INMD, and to design interventions supporting resilience as a protective factor for mental health promotion.

## Background

Italy was the first European nation facing the outbreak of severe acute respiratory syndrome coronavirus 2 (SARS-CoV-2) at the beginning of 2020 [1]. Lombardia was the first and most affected region in the country, particularly during the first wave of the pandemic. According to reports provided by the National Ministry of Health, by the beginning of November 2020 709335 persons had been infected throughout Italy, and 38826 of them had died; Lombardia heavily contributed to these national figures, with 204351 infection cases and 17589 deaths [2]. INMD was supposed to represent a risk factor for a more severe course and outcome of SARS-CoV-2 infection, due to the cardiac and respiratory complications, wheelchair dependency and immobility that characterize this kind of pathology. Additionally, persons diagnosed with INMD during the lockdown phases of the pandemic experienced discontinuation of care and treatments, being thus exposed to the risk of worsening health conditions, as well as exacerbation of physical and mental symptoms, especially anxiety, leading to a vicious circle of increased management concerns [3]. Overall, the pandemic-related restrictions posed two types of challenges to persons diagnosed with INMD and their family caregivers: at the psychological level, the exposure to emotional distress related to daily routine disruption, fear of contagion, and social life restrictions; at the physical health level, the reduction or interruption of both direct contacts with the reference healthcare centers and home care support activities.

### The investigation of mental health among persons with neuromuscular diseases

INMD comprises heterogeneous typologies of disorders, the most common being muscular dystrophies (MD). MD include different forms, such as Duchenne MD (DMD), Becker MD (BMD), limb-girdle MD (LGMD), facioscapulohumeral dystrophy (FSHD), and Myotonic dystrophy type 1 (DM1). All are characterized by progressive loss of muscle strength and a worsening ability to perform functional activities of daily living. They are also characterized by common psychosocial challenges (physical, social, and emotional), changing across each different stage of the disease [4].

To the best of our knowledge, research on the prevalence of psychopathology in INMD is still limited. The available evidence primarily concerns the association of INMD with emotional and behavioral difficulties. A recent study highlighted a high prevalence of internalizing problems (Anxious/Depressed, Withdrawn, Somatic Complaints), 18% and 24,6% among young adults and children, respectively [5]. The finding regarding children is consistent with those reported in two previous studies [6, 7]. Recent reviews were also focused on the prevalence of psychopathology in different types of dystrophies, showing a higher prevalence of anxiety and depression in patients with DMD (24% and 11%, respectively) and BMD (25% and 7%, respectively) [8] and higher rates of depression in patients with DM1 [9]. Despite the research convergence on the prevalence of internalizing problems in INMD patients, controversial results were obtained concerning externalizing problems (Aggressive Behavior, Rule-Breaking Behavior, Intrusive). It is, however, important to note that most of the studies mentioned above were based on caregivers’ perception of the emotional and behavioral features of their children. Studies relying on data directly obtained from youth and adults with INMD, as well as from their caregivers, highlighted that the former have a more positive perception of their own psychological status, compared to their caregivers [5, 6].

This possible bias in self-assessment and/or peer-assessment is even more evident as concerns the investigation of dimensions of positive psychological functioning among persons with INMD. In the last three decades, increasing efforts have been devoted to the development of mental health models comprising positive indicators of affective, cognitive, motivational and social well-being [10–13]. In line with the WHO’s definition of health as a state of well-being rather than absence of pathology, the assessment of positive dimensions of well-being across the most diverse populations, including persons in suboptimal health conditions, highlighted their usefulness for designing interventions aimed at promoting individual well-being beyond reducing symptomatology. Moreover, several studies conducted in this domain highlighted the role of resilience and social support as two relevant assets contributing to positive mental health under adverse circumstances.

The importance of treating patients not only by trying to lower negative symptomatology but also to promote well-being and improve resilience has been repeatedly highlighted [4]. Resilience can be defined as “the capacity of a system to adapt successfully to disturbances that threaten the viability, function, or development of the system” [14]. It represents a multidimensional and dynamic process emerging from the interaction among individual factors (genetic characteristics and personality traits, as well as acquired abilities and competences) and social-environmental factors (such as family functioning and social support) [15]. Two different approaches were adopted to conceptualize and operationalize resilience: the first one is based on the identification and measurement of personal and contextual resources supporting adaptive adjustment; the second one is focused on the direct assessment of the level of an individual’s ability to successfully cope with adversity. Both approaches led to the development of psychometrically solid instruments to assess resilience [16]. In samples derived from the general population, resilience was positively associated with well-being and negatively associated with depression [17, 18].

Social support refers to the perceived availability of friends, family members and significant others as sources of instrumental and psychological support in case of need. In most studies, perceived social support was positively associated with well-being and negatively associated with affective disorders and distress [19, 20].

Among persons with INMD, resilience is positively associated with age; evidence was collected about its role as a mediator in the association between secondary symptoms (e.g., fatigue, pain) and perceived quality of life [21] and as a predictor of satisfaction with social roles. Mixed results were instead obtained concerning its association with physical functioning [22, 23]. Both resilience and perceived social support were negatively associated with affective disorder symptoms [21, 24–26].

### The impact of the COVID-19 pandemic on mental health

Since 2020, several studies have been devoted to the investigation of the negative consequences of the pandemic on citizens’ mental health. In the general population, affective disorder symptoms increased slightly in the early phase of the pandemic and returned to prepandemic levels by mid-2020, whereas their levels showed a higher increase among individuals with suboptimal physical health conditions [27]. Although fewer studies have investigated well-being and positive mental health [27], recurrent evidence has been obtained regarding the protective role of social support and resilience during the pandemic [28–33].

Studies conducted among persons with different chronic diseases highlighted that the lifestyle changes imposed by pandemic-related restrictions put a strain on their mental health. Nevertheless, participants who experienced fewer changes also reported higher levels of resilience and lower levels of stress, depression and anxiety [34]. Regarding social support, partnered persons with chronic diseases reported lower support from their friends and family compared with a control group [35]. Similar findings emerged among adults with multiple sclerosis [36], who perceived lower social support and higher stress and were depressed in higher proportions than a control group. These negative symptoms could, however, predate the pandemic, as persons with multiple sclerosis often experience difficulties in their social life and mental health [37]. Research conducted during the pandemic on the association between social support and psychopathological outcomes in samples including participants with a broad spectrum of chronic illnesses provided mixed findings [38, 39], most likely related to the heterogeneous clinical conditions of the participants.

As specifically concerns persons with INMD, a survey conducted by the Italian Myology Association (AIM) in the first months of the pandemic explored the impact of the substantial rearrangements in the services provided to patients on their quality of life. Thirty-one AIM-affiliated clinical centers across the country took part in the study. Overall, the findings highlighted the disruption caused by the COVID-19 pandemic in clinical and support services for patients. More specifically, 22% of the centers had postponed the administration of drugs usually taking place in hospitals, and 57% had suspended diagnostic activities and treatments such as physiotherapy, nursing care and psychological support. Rehabilitation activities were suspended in 93% of the centers. In order to meet patients’ needs under these problematic circumstances, several centers kept outpatient visits active for emergencies, while simultaneously implementing remote and telemedicine activities [3].

To the best of our knowledge, no studies have been conducted during the pandemic to investigate resilience and social support among persons with INMD. Considering that evidence obtained from persons with widely different chronic conditions might not accurately represent the experience of individuals diagnosed with these specific disorders, the present study was aimed to investigate positive and negative aspects of the experience reported by persons with INMD during the pandemic, focusing on (a) levels of resilience and perceived social support; (b) prevalence and intensity of psychopathological symptoms, and (c) the relationship between positive and psychopathological dimensions.

## Materials and methods

### Participants and procedures

Data were collected between Spring and Fall 2020 through the joint collaboration of Scientific Institute IRCCS “E. Medea”, IRCCS “Ospedale San Raffaele”, and IRCCS “Mondino Foundation” Pavia. This cross-sectional observational study was approved by the local Ethics Committees. In accordance with the Helsinki Declaration, records of all patients with INMD were inspected, and eligible participants were identified based on the following inclusion criteria:

1. Carrying a defined diagnosis of INMD according to internationally recognized criteria [40–44]. The clinical diagnosis had been formulated by an expert neurologist working in the neuromuscular field for over 15 years. Moreover, all patients had a confirmed biochemical and genetic diagnosis (i.e. mutations in the dystrophin gene for patients with Duchenne and Becker muscular dystrophy, mutations in calpain3, dysferlin and beta sarcoglycan for patients with Limb Girdle muscular dystrophy, deletion in the repeated sequences in D4Z4 region for FSHD and CTG triplets amplification in the DMPK gene for patients with Myotonic dystrophy, mutations in the LMNA for congenital muscular dystrophies, duplication in the PMP22 gene in the patient with Charcot Marie Tooth type 1 A).
2. Age range between 15 and 59 years
3. Signature of informed consent

A total of 99 eligible participants were identified; all the materials, including a brief description of the study, an informed consent form and the questionnaires, were sent to them via e-mail. All participants or parents/legal tutors of minors were invited to sign the informed consent; participants were asked to complete the online version of the questionnaires and send them back. The 99 eligible individuals were initially contacted via e-mail. Among them, 25 never replied to the e-mail, whereas 15 provided their consent to participate but they did not complete the questionnaires. These were the reasons for excluding 40 persons from the final sample. No further exclusion criteria (age, diagnosis, gender, other clinical and sociodemographic features) were adopted. Valid data were obtained from 59 participants; they completed the questionnaires and underwent clinical evaluation either in hospital (when possible, based on the pandemic phase and related restrictions) or through a televisit. Data were pseudonymized for analysis.

### Measures

#### Sociodemographic and clinical features

Participants provided information about their age, sex, occupational or student status, educational level, family composition and house characteristics. They also answered questions concerning their diagnosis and their level of autonomy. Collected sociodemographic information are reported in Table 1.

**Table 1:** Sociodemographic characteristics of the three groups

	DMD	DM1	Slowly progressive neuromuscular disorders
**N° Participants**	15 (Male = 15; Female = 0)	15 (Male = 4; Female = 11)	29 (Male = 23; Female = 6)
**Age**	15–36 (*M* = 23.80, *SD* = 6.81)	24–59 (*M* = 39.47, *SD* = 11.15)	20–57 (*M* = 39.52, *SD* = 11.92)
**More than 2 family members at home**	13 (87%)	9 (60%)	19 (65%)
**House characteristics**: **Single-family house** **Apartment**	3 (20%) 12 (80%)	4 (27%) 11 (73%)	4 (14%) 25 (86%)
**Education (High School Graduate)**	8 (53%)	8 (53%)	22 (76%)
**Worker**	0	5	17
**Student**	7	1	2

M = mean; SD = standard deviation; DMD = Duchenne muscular dystrophy; DM1 = Myotonic dystrophy type 1; Apartment (duplex house, Large complex house, Three-family house)

#### Multidimensional Scale of Perceived Social Support (MSPSS) [45–47]

The MSPSS is a 12-item self-report scale measuring perceived social support on a 6-point Likert scale (coded as 1 = very strongly disagree; 7 = very strongly agree). The scale has a three-factor structure, with each factor respectively measuring perceived social support from friends, family and a significant other. The Italian version showed excellent internal reliability for the total scale (α = 0.91) and its subscales (friends α = 0.90; family α = 0.92; significant other α = 0.93).

#### Resilience Scale for Adults (RSA) [48, 49]

The RSA is a 33-item self-report scale measuring resilience on a 7-point semantic differential scale; each item has a positive and a negative attribute at each end of the scale continuum. Positive attributes are positioned to the right for half of the items to reduce acquiescence biases. The RSA has a six-factor structure: four factors concern individual characteristics (Perception of Self, Planned Future, Social Competence, Structured Style), one refers to the family environment (Family Cohesion) and one to social networks (Social Resources). The Italian version [48] showed good internal reliability for the whole scale (α = 0.90) and for five of the subscales (0.75 < α < 0.82), except for Structured Style (α = 0.34).

#### Achenbach System of Empirically Based Assessment (ASEBA) [50–52]

The ASEBA questionnaires provide a description of children’s and adults’ behavioral and emotional profiles during the previous 6 months through accounts provided by the caregiver (Child Behavior Checklist, CBCL/6–18; Adult Behavior Checklist, ABCL/18–59) and/or by the participants themselves (Youth Self Report, YSR/11–18; Adult Self Report, ASR/18–59) [50, 51]. ASEBA is a scale used internationally to measure psychopathological symptoms. It has several advantages: it is easy to use; it represents an optimal instrument for identifying intervention needs and assessing behavioral, emotional and social problems. It also has good predictive accuracy. This tool was used with a variety of populations, including persons with INMD [5, 6, 53–55]. In the present study, the ASEBA’s self-report questionnaires (YSR and ASR) were used. They comprise 126 and 113 items, respectively, rated on a 3-point scale: 0 = Not True; 1 = Somewhat or Sometimes True; 2 = Very True or Often True. Items are grouped into 8 syndrome scales: anxious/depressed, withdrawn, somatic complaints, thought problems, attention problems, aggressive behavior, and rule-breaking behavior. The label of each syndrome reflects the specific typology of problems characterizing the syndrome. The total score for each syndrome scale is computed by summing the value of the individual item. The ASEBA instrument has multicultural norms. For all scale scores, T-scores are provided, which represent standardized values based on age and sex norms of the Italian population, displayed in a normal distribution with a mean of 50 and a standard deviation of 10 in absolute values [52]. Following the literature, for the 8 syndrome scales of ASEBA T scores above 69 are in the clinical range (indicating that the participant reported enough problems to be of clinical concern); T scores between 65 and 69 are in the borderline range (high enough to be of concern but not so clearly deviant as scores in the clinical range); T scores below 64 are in the normal range [52].

### Statistical analyses

Statistical analyses were conducted using IBM SPSS 28 [56]. Mean scores were computed for each variable. Pearson’s correlation coefficients were calculated between indicators of well-being (resilience and perceived social support) and emotional and behavioral symptoms.

Mann Whitney U tests were used to detect differences in well-being-related variables between participants scoring above or below psychopathology thresholds, as well as differences in well-being-related variables and emotional and behavioral symptoms according to participants’ self-reported autonomy, assessed as a dichotomous variable (yes/no).

Mann Whitney U tests were employed after checking for Student’s t-test assumptions. Levene tests were conducted to assess variance homogeneity, which was always respected, except when comparing withdrawal symptoms according to participants’ autonomy. Shapiro-Wilk tests showed normality violations for all variables, except for total resilience and its two subscales Perception of self and Planned future. Therefore, Mann Whitney U tests were performed instead of t-tests. In order to assure comparability, Mann Whitney U tests were also employed to analyze variables that respected assumptions.

Hierarchic logistic regressions were conducted to examine whether resilience and physical diagnosis predicted psychopathology better than diagnosis alone. Goodness of fit for regression models was calculated using Hosmer-Lemeshow test.

## Results

### Participants

Analyses were conducted on the data collected by the 59 participants who had completed all the questionnaires (71.2% M, age range 15–59, M age = 35.,51, SD = 12,54). They were divided in three subgroups in relation to diagnosis and disease evolution (for the group of patients with Duchenne muscular dystrophy and for the ones with Myotonic dystrophy, diagnosis and disease evolution were quite homogeneous; a third group was composed by patients sharing a slowly progressive evolution of the disease, however based on a heterogeneous genetic background). The three groups were composed as follows: (1) 15 participants with Duchenne muscular dystrophy (DMD), (2) 15 with Myotonic dystrophy type 1 (or Steinert Disease), and (3) 29 with slowly progressive neuromuscular disorders, including 14 cases of Limb-Girdle muscular dystrophy, 8 with Becker muscular dystrophy, 4 with congenital myopathies, 2 with facioscapulohumeral muscular dystrophy, and 1 with Charcot-Marie-Tooth disease.

Three groups were characterized by:

1. Duchenne muscular dystrophy: 15 male participants, aged 15–36 (*M* = 23.80, *SD* = 6.81), 6 lack of autonomy in daily living, 14 wheelchair-bound, 9 with cardiomyopathy, 7 with respiratory insufficiency requiring mechanical non-invasive ventilation.

2) Myotonic dystrophy type 1: 15 participants, aged 24–59 (*M* = 39.47, *SD* = 11.15), 2 lack of autonomy in daily living, 1 wheelchair-bound, 5 with cardiac disorders and 4 with respiratory insufficiency requiring mechanical non-invasive ventilation.

3) Slowly progressive neuromuscular disorders genetically defined: 29 participants, aged 20–57 (*M* = 39.52, *SD* = 11.92), 7 lack of autonomy in daily living, 13 non ambulant, 9 with cardiac disorders and 3 with respiratory insufficiency requiring non-invasive mechanical ventilation (see Table 2).

**Table 2:** Clinical features of the three groups

	DMD	DM1	Slowly progressive neuromuscular disorders
**Ambulant (N/%)**	1 (7%)	14 (93%)	16(55%)
**Wheelchair bound (N/%)**	14 (93%)	1 (7%)	13 (45%)
**Cardiomyopathy (N/%)**	9 (60%)	5 (33%)	9 (31%)
**Respiratory insufficiency (N/%)**	7 (46%)	4 (27%)	3 (10%)
**Autonomy in daily living (ADL)**	2 (13%)	2 (13%)	7 (24%)

M = mean; SD = standard deviation; DMD = Duchenne muscular dystrophy; DM1 = Myotonic dystrophy type 1

### Well-being and psychopathology dimensions

As Table 3 shows, internal reliability was adequate for MSPSS and RSA, as well as for their subscales (0.708 < α < 0.948), except for the “Structured Style” and “Social Competence” dimensions of RSA, which were thus excluded from subsequent analyses.

**Table 3:** Scale reliability

Variables	Cronbach’s α
MSPSS total	0.908
MSPSS friends	0.948
MSPSS family	0.901
MSPSS significant other	0.874
RSA total	0.874
PS Perception of Self	0.708
PF Planned Future	0.834
SC Social Competences	0.536
FC Family Cohesion	0.853
SS Structured Style	0.565
SR Social Resources	0.713
**ASEBA subscales**	
Anxious/Depressed	0.833
Withdrawn	0.806
Somatic	0.704
Thought	0.581
Attention	0.774
Aggressive	0.784
RuleBreaking	0.580

MSPSS: Multidimensional Scale of Perceived Social Support; RSA: Resilience Scale for Adults; PS: Perception of Self; PF: Planned Future; SC: Social Competences; FC: Family Cohesion; SS: Structured Style; SR: Social Resources; ASEBA: Achenbach System of Empirically Based Assessment

For the ASEBA subscales, the internal reliability was calculated only on the Adult Self Report (ASR) because only 4 participants filled the Youth Self Report (YSR). The internal reliability for ASR subscales was adequate (0.704 < α < 0.833), except for the “Thought Problems” and “Rule-Breaking Behavior” subscales, leading to their exclusion from the subsequent analyses.

As reported in Table 4, perceived social support and resilience mean scores were all above the midpoint of the scales, while the prevalence of psychopathological symptoms was low. The latter finding was not surprising, as the majority of participants scored below the pathology threshold (Table 5).

**Table 4:** Mean scores and standard deviations of resilience and social support in the three groups

	Total sample (*N* = 59)		DMD (*N* = 15)		Steinert disease (*N* = 15)		Slowly progressive (*N* = 29)	
	M	SD	M	SD	M	SD	M	SD
**MSPSS total**	5.53	1.16	5.75	0.90	5.54	1.22	5.41	1.27
**MSPSS friends**	4.94	1.69	5.03	1.38	4.99	1.70	4.86	1.88
**MSPSS family**	5.88	1.32	6.25	0.85	5.60	1.65	5.84	1.33
**MSPSS significant other**	5.76	1.37	5.97	0.93	6.02	1.23	5.52	1.60
**RSA total**	5.32	0.77	5.48	0.70	4.93	0.73	5.44	0.77
**RSA PS**	5.34	1.03	5.39	0.90	4.81	1.07	5.59	1.00
**RSA PF**	4.14	1.59	4.03	1.31	3.53	1.55	4.51	1.69
**RSA FC**	5.52	1.34	5.99	0.91	5.39	1.58	5.34	1.38
**RSA SR**	5.95	0.86	6.14	0.67	5.76	1.06	5.94	0.84
**Anxiety/Depression**	56.37	6.38	57.20	6.25	56.80	6.84	55.72	6.36
**Withdrawn**	56.31	7.58	55.93	9.14	57.87	8.85	55.69	6.02
**Somatic**	56.02	6.19	55.27	4.28	57.60	6.84	55.59	6.69
**Attention**	56.15	6.38	54.40	3.66	61.67	7.42	54.21	5.31
**Aggressive**	54.83	5.71	52.73	4.20	57.20	6.09	54.69	5.89

MSPSS: Multidimensional Scale of Perceived Social Support; RSA: Resilience Scale for Adults; PS: Perception of Self; PF: Planned Future; FC: Family Cohesion; SR: Social Resources; DMD: Duchenne Muscular Dystrophy; M: Mean; SD: Standard Deviation

**Table 5:** Frequency and percentage distribution of participants above and below the psychopathology threshold in the three groups

		Total sample (*N* = 59)	DMD (*N* = 15)	Steinert disease (*N* = 15)	Slowly progressive (*N* = 29)
**Anxiety/** **Depression**	Above threshold N (%)	17 (28.81)	7 (46.67)	5 (33.33)	5 (17.24)
	Below threshold N (%)	42 (71.19)	8 (53.33)	10 (66.67)	24 (82.76)
**Withdrawn**	Above threshold N (%)	12 (20.34)	3 (20)	4 (26.67)	5 (17.24)
	Below threshold N (%)	47 (79.66)	12 (80)	11 (73.33)	24 (82.76)
**Somatic**	Above threshold N (%)	14 (23.73)	1 (6.67)	6 (40)	7 (24.14)
	Below threshold N (%)	45 (76.27)	14 (93.33)	9 (60)	22 (75.86)
**Attention**	Above threshold N (%)	12 (20.34)	0 (0)	9 (60)	3 (10.34)
	Below threshold N (%)	47 (79.66)	15 (100)	6 (40)	26 (89.66)
**Aggressive**	Above threshold N (%)	13 (22.03)	2 (13.33)	6 (40)	5 (17.24)
	Below threshold N (%)	46 (77.97)	13 (86.67)	9 (60)	24 (82.76)

Well-being dimensions and emotional and behavioral symptoms varied according to participants’ perceived autonomy vs. lack of autonomy in daily living. Participants perceiving complete dependence reported significantly higher levels of anxiety/depression (U = 143, *p* = 0.015). They also scored significantly lower on the Perception of Self dimension of resilience (U = 147, *p* = 0.019). No differences were instead detected in perceived social support.

### The relationship between well-being and psychopathology dimensions

As shown in Table 6, we calculated Pearson’s correlation coefficients between psychopathological symptoms and well-being related variables. We interpreted results according to Cohen (1988) guidelines. Associations were considered small when the correlation coefficient was 0.10 ≤ *r* ≤ 0.29, moderate when 0.30 ≤ *r* ≤ 0.49, and strong when the coefficient was *r* = 0.50 or larger [57]. Anxious and depressive symptoms and Attention symptoms were moderately and negatively correlated with total resilience and its two subdimensions Perception of Self and Planned future. Withdrawal symptoms showed a moderate and negative correlation with resilience and small negative correlations with Perception of Self, Family Cohesion, Social Resources; the Withdrawn scale also displayed small negative correlations with perceived social support (MSPSS) and its subdimension of support from a significant other. Lastly, Aggressive symptoms had a moderate and negative correlation with Perception of Self.

**Table 6:** Pearson’s correlations (r) between symptoms and positive mental health dimensions

		Anxious/ Depressed	Withdrawn	Somatic	Attention	Aggressive
**RSA total**		− 0.396**	− 0.347**	− 0.143	− 0.365**	− 0.216
**RSA PS**		− 0.479**	− 0.291*	− 0.224	− 0.478**	− 0.312*
**RSA PF**		− 0.350**	− 0.117	− 0.239	− 0.433**	− 0.215
**RSA FC**		− 0.182	− 0.257*	0.040	0.039	− 0.092
**RSA SR**		− 0.227	− 0.260*	− 0.049	− 0.072	− 0.169
**MSPSS total**		− 0.095	− 0.276*	0.071	0.194	− 0.068
**MSPSS friends**		− 0.030	− 0.217	0.077	0.243	0.005
**MSPSS family**		− 0.057	− 0.165	0.039	0.141	− 0.025
**MSPSS s.o.**		− 0.149	− 0.275*	0.048	0.056	− 0.154

** *p* < .01; * *p* < .05

RSA: Resilience Scale for Adults; PS: Perception of Self; PF: Planned Future; FC: Family Cohesion; SR: Social Resources; MSPSS: Multidimensional Scale of Perceived Social Support; s. o.: significant other. The most significant results are highlighted in bold type

As highlighted in Table 7, significant differences in both the resilience total score and in the scores of Perception of Self and Planned Future were detected between participants scoring above and below the thresholds for most psychopathological symptoms, except somatic symptoms. No differences instead emerged in perceived social support.

**Table 7:** Comparison of total and subscale resilience levels of participants above and below the psychopathology threshold

Variables	RSA dimensions	Above threshold M	Below threshold M	U	*p*
**Anxiety/** **Depression** (N above = 17; N below = 42)	RSA	4.93	5.48	204	**0.011**
	PS	4.60	5.64	152	**< 0.001**
	PF	3.44	4.42	235	**0.041**
	FC	5.40	5.56	329	0.638
	SR	5.72	6.04	282	0.208
**Withdrawn** (N above = 12; N below = 47)	RSA	4.81	5.45	143	**0.009**
	PS	4.72	5.50	161	**0.022**
	PF	3.75	4.24	227	0.299
	FC	4.89	5.68	173	0.039
	SR	5.61	6.03	205	0.148
**Somatic** (N above = 14; N below = 45)	RSA	5.24	5.34	299	0.776
	PS	5.18	5.39	280	0.532
	PF	3.64	4.29	240	0.180
	FC	5.54	5.51	308	0.907
	SR	5.90	5.96	287	0.617
**Attention** (N above = 12; N below = 47)	RSA	4.74	5.47	126	**0.003**
	PS	4.47	5.56	107	**< 0.001**
	PF	3.21	4.38	164	**0.026**
	FC	5.33	5.56	244	0.472
	SR	5.65	6.02	208	0.165
**Aggressive** (N above = 13; N below = 46)	RSA	4.93	5.43	193	0.052
	PS	4.67	5.53	56	**0.009**
	PF	3.25	4.39	189	**0.044**
	FC	5.29	5.58	261	0.485
	SR	5.73	6.01	243	0.304

RSA: Resilience Scale for Adults; PS: Perception of Self; PF: Planned Future; FC: Family Cohesion; SR: Social Resources. The most significant results are highlighted in bold type

Finally, we investigated whether resilience and INMD diagnosis could predict psychopathology better than diagnosis alone. Hierarchical logistic regression models with each symptom as a dependent variable were performed. The dependent variables were dichotomous (being above or below the threshold for psychopathological symptoms). The independent variables were continuous (resilience and its sub-dimensions) or categorical (diagnosis). Diagnosis was dummy coded, with Slowly progressive being chosen as the baseline category. In the first block of the hierarchical logistic regression, we used the INMD diagnosis as the independent variable and a categorical variable (being above or below the threshold for a psychopathological symptom) as the dependent variable.

In Block 2 resilience was added as a second independent variable. As whole, resilience increased the predictive power of the model for anxious/depressive symptoms (Nagelkerke index from *R*^2^_N_ = 0.102 to *R*^2^_N_ = 0.264) and attention problems (from *R*^2^_N_ = 0.454 to *R*^2^_N_ = 0.536).

As shown in Table 8, DMD (B = 1.748, S.E.=0.794, *p* =0 .028, OR = 5.744), but not Steinert disease (B = 0.388, S.E.=0.801, *p* = 0.628, OR = 1.474), significantly predicted anxious/depressive symptoms. Participants with higher resilience levels were significantly less prone to report anxious/depressive symptoms (B=-1.264, S.E.=0.501, *p* = 0.012, OR = 0.283). We calculated goodness of fit using Hosmer-Lemeshow test, which resulted significant (*p* = 0.008) for the logistic regression using diagnosis and resilience as independent variables, and anxiety/depression as dependent variable. Regarding resilience dimensions, Perception of Self was a significant predictor of anxiety/depression symptoms (B=-1.590, S.E.=0.522, *p* =0 .002, OR = 0.204) and goodness of fit for this regression was acceptable (*p* = 0.217).

**Table 8:** Final logistic regression model for anxiety/depression symptoms

	B	S.E.	*p*	OR
**Diagnosis** *(slowly progressive)* DMD	1.748	0.794	**0.028**	5.744
Steinert disease	0.388	0.801	0.628	1.474
**RSA total**	-1.264	0.501	**0.012**	0.283

RSA: Resilience Scale for Adults; DMD: Duchenne Muscular Dystrophy; S. E: Standard Error; OR: Odds Ratio. Significant results are highlighted in bold type

As shown in Table 9, resilience was the only predictor of withdrawal symptoms (B=-1.266, S.E.= 0.532, *p* = 0.017, OR = 1.046), since DMD (B = 0.315, S.E.= 0.866, *p* =0 .716, OR = 1.371) and Steinert (B = 0.019, S.E.= 0.836, *p* =0 .982, OR = 1.019) resulted non-significant. Goodness of fit was acceptable (*p* = 0.637).

**Table 9:** Final logistic regression model for withdrawal symptoms

	B	S.E.	*p*	OR
**Diagnosis** *(slowly progressive)* DMD	0.315	0.866	0.716	1.371
Steinert disease	0.019	0.836	0.982	1.019
**RSA total**	-1.266	0.532	**0.017**	0.282

RSA: Resilience Scale for Adults; DMD: Duchenne Muscular Dystrophy; S. E: Standard Error; OR: Odds Ratio. Significant results are highlighted in bold type

Neither INMD diagnosis nor resilience represented significant predictors for somatic (DMD: B=-1.496, S.E.=1.123, *p* =0 .183, OR = 0.224; Steinert: B = 0.763, S.E.= 0.720, *p* =0 .289, OR = 2.145; RSA: B = 0.045, S.E.=0.437, *p* =0 .918, OR = 1.046) (Table 10) and aggressive symptoms (DMD: B=-0.265, S.E.=0.922, *p* = 0.773, OR = 0.767; Steinert: B = 0.858, S.E.= 0.755, *p* =0 .256, OR = 2.358; RSA: B=-0.743, S.E.=0.473, *p* =0 .117, OR = 0.476) (Table 11). Goodness of fit was acceptable for both regressions (*p* = 0.722 for somatic complaints, *p* =0 .637 for aggressive behavior).

**Table 10:** Final logistic regression model for somatic complaints

	B	S.E.	*p*	OR
**Diagnosis** *(slowly progressive)* DMD	-1.496	1.123	0.183	0.224
Steinert disease	0.763	0.720	0.289	2.145
**RSA total**	0.045	0.437	0.918	1.046

RSA: Resilience Scale for Adults; DMD: Duchenne Muscular Dystrophy; S. E: Standard Error; OR: Odds Ratio. Significant results are highlighted in bold type

**Table 11:** Final logistic regression model for aggressive behaviors

	B	S.E.	*p*	OR
**Diagnosis** *(slowly progressive)* DMD	-0.265	0.922	0.773	0.767
Steinert disease	0.858	0.755	0.256	2.358
**RSA total**	-0.743	0.473	0.117	0.476

RSA: Resilience Scale for Adults; DMD: Duchenne Muscular Dystrophy; S. E: Standard Error; OR: Odds Ratio. Significant results are highlighted in bold type

Lastly, suffering from Steinert disease was the only predictor of attention problems (B = 2.339, S.E.=0.858, *p* = 0.006, OR = 10.367). As shown in Table 12, DMD (B=-18.942, S.E.=9917.607, *p* =0. 998, OR = 0.000) and resilience (B=-1.260, S.E.=0.649, *p* =0 .052, OR = 0.284) did not significantly predict attention problems. Goodness of fit test was not significant (*p* =0 .169), indicating acceptable goodness of fit.

**Table 12:** Final logistic regression model for attention problems

	B	S.E.	*p*	OR
**Diagnosis** *(slowly progressive)* DMD	-18.942	9917.607	0.998	0.000
Steinert disease	2.339	0.858	**0.006**	10.367
**RSA total**	-1.260	0.649	0.052	0.284

RSA: Resilience Scale for Adults; DMD: Duchenne Muscular Dystrophy; S. E: Standard Error; OR: Odds Ratio. Significant results are highlighted in bold type

## Discussion

This study was aimed to jointly explore, for the first time and in the context of the COVID-19 pandemic, the relationship between psychopathological symptoms and the well-being dimensions of resilience and social support among people with INMD. Therefore, the main focus was to describe the experiences of well-being and ill-being reported by participants with a diagnosis of INMD during the pandemic. As specifically concerns the assessment of resilience, consistent with the view based on the identification and measurement of personal and contextual resources supporting adaptive adjustment, the Resilience Scale for Adults was deemed as an adequate instrument, as it shows good psychometric properties and cross-cultural consistency, it offers exhaustive coverage of the major personal, family, and social resources contributing to positive adjustment, and it can inform clinical interventions, by providing information on individual and contextual dimensions to be supported or developed to help individuals successfully cope with stressful conditions [16]. Two resilience subscales (Structured Style and Social Competence) showed low internal consistency, and therefore were discarded from analysis. As concerns Structured Style, this finding is aligned with evidence obtained in other samples across nations, suggesting its peculiarity as a cultural feature [58–60]. The low internal consistency of the Social Competence subscale can be instead related to the serious mobility limitations, health-related needs and constraints in daily functioning which prevent most participants from autonomously experimenting with the search and development of social connections outside the family and the closer relational network.

On average, participants showed good levels of resilience and perceived social support. Resilience mean scores were above the midpoint for all the subscales. The slightly lower scores reported in the Planned Future subscale could be related to the uncertainty perceived by the participants, facing increasing limitations and health problems imposed by a progressive disease. This disease-related feeling may have been amplified during the pandemic, in which perceived uncertainty was widely documented in the general population [61, 62]. Participants’ perception of social support was primarily related to their family. This finding can be related to cultural aspects characterizing Italian families, still connected by strong bonds of reciprocity and mutual aid, including the care of relatives with physical and mental disorders [63]. For persons with INMD, the pandemic might have even reinforced perceived family support, as restrictions led families to spend more time together.

Only a minority of participants experienced clinically relevant psychopathological symptoms (28.81% for anxiety/depression). The participants in our study self-reported a higher level of anxiety/depression, in contrast to the higher level of internalizing problems referred by caregivers in previous studies (28% vs. 24%). Therefore, in future studies it would be interesting to identify demographic, clinical and/or environmental variables that could contribute to discrepancies between different evaluators.

Psychopathological dimensions and resilience were negatively correlated, while social support was inversely associated with withdrawal symptoms only. In line with another study involving persons with chronic conditions [38], social support and psychopathological outcomes were not significantly correlated with each other.

Furthermore, most associations between psychopathology symptoms and resilience involved two dimensions of resilience related to the individual (Perception of Self and Planned Future).

In line with previous studies [6–8], participants with DMD were more likely to suffer from anxious and depressive symptoms, while those with DM1 had higher odds of experiencing attention problems. Resilience emerged as a predictor of lower anxious-depressive symptoms. These findings suggest that the promotion of resilience could contribute to at least buffering some manifestations of psychopathology, as demonstrated in a recent review, which supports the role of individual and relational resilience factors as protective resources that produce transdiagnostic effects in children and youth at risk for psychopathology [14].

Overall, the results from the present study suggest that it is possible to perceive well-being and mobilize positive psychological resources under adverse circumstances, such as living during a pandemic emergency while being diagnosed with INMD. Nevertheless, the cross-sectional design of the study did not allow for detecting psychological changes related to the emergency condition of the COVID-19 pandemic, which had a widespread negative emotional impact across populations. Remote online support interventions were implemented and proved to be helpful in counterbalancing social isolation, particularly in frail groups such as the elderly [64]. Unfortunately, to date no evidence is available on this issue as concerns persons with INMD. Research was only conducted to investigate the effectiveness of online physiotherapy for patients with INMD, showing worse outcomes compared to in-person treatments [65].

Indeed, the health conditions of persons with INMD must be consistently monitored by professionals. The support provided by clinical centers is key to fostering their physical and mental health. Psychological support might be especially beneficial for those who are completely dependent on their caregivers; in the present study, they reported a less resilient perception of self and more anxious and depressive symptoms.

Our findings further highlight the importance of investigating positive resources, in order to identify areas of functioning that can be supported in persons with suboptimal mental health. Psychosocial interventions aimed at developing individual resilience may help foster well-being and protect from psychopathology, by focusing on the promotion of an adequate perception of self and a more proactive management of future perspectives. Some interventions in this direction were addressed to children and adolescents with DMD and their families [66, 67]. We emphasize the importance of psychological and social support for people with INMD and their family members or caregivers, in order to improve quality of life and to reduce fatigue [4]. Further research is however needed to identify evidence-based programs to promote resilience in persons with INMD.

### Strengths and limitations

Both strengths and limitations characterize this study.

To our knowledge, this is the first study to investigate social support, resilience and their relationship with psychopathology in INMD during the COVID-19 pandemic.

At the same time, the cross-sectional design allows for detecting only associations among variables, thwarting any causal interpretation of these relationships. Moreover, the small sample size and the lack of a control group from the general population do not allow for generalization of the findings. Considering the sample size, each analysis included two predictors at most, in order to avoid the reduction of statistical power. Another limitation of the study is the uneven sample distribution according to gender - which reflects the higher prevalence of neuromuscular disorders among men - and diagnosis. It is, however, worth noting that participants in this study belong to a very specific group of people diagnosed with rare diseases; moreover, data were collected during a period of health emergency, in which most healthcare structures were under pressure, making the involvement of potential study participants more problematic. Another limitation concerns the psychopathological dimensions of anxiety and depression, which were examined jointly through the ASEBA scales.

Finally, the lack of a control group does not allow to evaluate whether persons with INMD showed higher rates psychopathology symptoms compared to the general population during the COVID-19 pandemic. However, multiple studies have documented an increase in internalizing symptoms among the general population, especially younger generations [68–70]. In addition, this is not a follow-up study, and it is thus impossible to identify participants’ changes in well-being and psychopathology related to the pandemic outbreak.

Despite the above mentioned limitations, these findings open a new research avenue, suggesting the need for (a) longitudinal studies investigating the potential of resilience in supporting well-being and buffering psychopathology symptoms among persons with INMD; (b) prospective clinical studies with follow up and control groups, to evaluate the potential of interventions promoting resilience among persons with INMD; (c) the usefulness of online support interventions for persons with INMD in counterbalancing social isolation and promoting social competences; (d) the potential role of psychological support to promote higher resilience in INMD persons totally dependent on their caregivers.

## Conclusion

This study explores for the first time in the context of COVID-19 pandemic, the relationship between psychopathological symptoms and well-being dimensions of resilience assessed by RSA and social support assessed by MSPSS among people with INMD.

The results show that the participants had good levels of resilience and perceived social support during the pandemic; only a minority of them manifested clinically relevant psychopathological internalizing symptoms (anxiety and depression). The results also show that psychopathological dimensions are negatively correlated with resilience, in particular with two specific dimensions related to the individual: self-perception and planning for the future. No significant correlations instead emerged between psychopathological symptoms and social support, except for an inverse association with withdrawal symptoms. These data suggest that resilience is a predictor of lower anxiety-depressive symptoms; promoting it could help buffer some manifestations of psychopathology. For this reason, it is crucial to provide psychological support to all patients, particularly those who are completely dependent on their caregivers, who report lower perceived resilience and more anxiety and depressive symptoms, because it could lead to greater benefits. Psychological support should be geared not only to difficulties but also to the patient’s positive resources, in order to identify and validate areas of functioning and promote adequate self-perception. It is also worth emphasizing that, besides psychological support, social support is essential to improve the quality of life and daily experience of both patients and caregivers.

## Data Availability

The data supporting the findings of this study are available from the corresponding author upon reasonable request.

## Abbreviations

INMD: Inherited neuromuscular disorders
SARS-CoV-2: severe acute respiratory syndrome –coronavirus2
MD: Muscular dystrophies
DMD: Duchenne Muscular dystrophies
BMD: Becker Muscular dystrophies
LGMD: Limb-Girdle Muscular dystrophies
FSHD: Facioscapulohumeral dystrophy
DM1: Myotonic dystrophy type 1
WHO: World Health Organization
AIM: Italian Myology Association
MSPSS: Multidimensional Scale of Perceived Social Support
RSA: Resilience Scale for Adults
ASEBA: Achenbach System of Empirically Based Assessment
CBCL/6–18: Child Behavior Checklist
ABCL/18–59: Adult Behavior Checklist
YSR/11–18: Youth Self Report
ASR/18–59: Adult Self Report
PS: Perception of Self
PF: Planned Future
SC: Social Competences
FC: Family Cohesion
SS: Structured Style
SR: Social Resources
